# Supplementary material for: Daptomycin versus Vancomycin for the Treatment of Methicillin-Resistant Staphylococcus aureus Bloodstream Infection with or without Endocarditis: A Systematic Review and Meta-Analysis
Source: Antibiotics (Basel). 2021 Aug 21;10(8):1014. doi: 10.3390/antibiotics10081014 (PMC8389004; doi:10.3390/antibiotics10081014)
Supplement: Supplementary file 1 [file antibiotics-10-01014-s001.zip › antibiotics-1320451-supplementary.pdf]

Table S1. PRISMA 2020 (Preferred Reporting Items for Systematic Review and Meta-Analysis) checklist.

Section and Topic	Item #	Checklist item	Reported on page #
**TITLE**			
Title	1	Identify the report as a systematic review.	1
**ABSTRACT**			
Abstract	2	See the PRISMA 2020 for Abstracts checklist.	2
**INTRODUCTION**			
Rationale	3	Describe the rationale for the review in the context of existing knowledge.	2
Objectives	4	Provide an explicit statement of the objective(s) or question(s) the review addresses.	2
**METHODS**			
Eligibility criteria	5	Specify the inclusion and exclusion criteria for the review and how studies were grouped for the syntheses.	3-5
Information sources	6	Specify all databases, registers, websites, organisations, reference lists and other sources searched or consulted to identify studies. Specify the date when each source was last searched or consulted.	3-5, Figure 1
Search strategy	7	Present the full search strategies for all databases, registers and websites, including any filters and limits used.	3-5, Table S2
Selection process	8	Specify the methods used to decide whether a study met the inclusion criteria of the review, including how many reviewers screened each record and each report retrieved, whether they worked independently, and if applicable, details of automation tools used in the process.	3-5
Data collection process	9	Specify the methods used to collect data from reports, including how many reviewers collected data from each report, whether they worked independently, any processes for obtaining or confirming data from study investigators, and if applicable, details of automation tools used in the process.	3-5
Data items	10a	List and define all outcomes for which data were sought. Specify whether all results that were compatible with each outcome domain in each study were sought (e.g. for all measures, time points, analyses), and if not, the methods used to decide which results to collect.	3-5
	10b	List and define all other variables for which data were sought (e.g. participant and intervention characteristics, funding sources). Describe any assumptions made about any missing or unclear information.	3-5
Study risk of bias assessment	11	Specify the methods used to assess risk of bias in the included studies, including details of the tool(s) used, how many reviewers assessed each study and whether they worked independently, and if applicable, details of automation tools used in the process.	3-5
Effect measures	12	Specify for each outcome the effect measure(s) (e.g. risk ratio, mean difference) used in the synthesis or presentation of results.	3-5
Synthesis methods	13a	Describe the processes used to decide which studies were eligible for each synthesis (e.g. tabulating the study intervention characteristics and comparing against the planned groups for each synthesis (item #5)).	3-5
	13b	Describe any methods required to prepare the data for presentation or synthesis, such as handling of missing summary statistics, or data conversions.	3-5
	13c	Describe any methods used to tabulate or visually display results of individual studies and syntheses.	3-5
	13d	Describe any methods used to synthesize results and provide a rationale for the choice(s). If meta-analysis was performed, describe the model(s), method(s) to identify the presence and extent of statistical heterogeneity, and software package(s) used.	3-5
	13e	Describe any methods used to explore possible causes of heterogeneity among study results (e.g. subgroup analysis, meta-regression).	3-5
	13f	Describe any sensitivity analyses conducted to assess robustness of the synthesized results.	3-5
Reporting bias assessment	14	Describe any methods used to assess risk of bias due to missing results in a synthesis (arising from reporting biases).	3-5
Certainty assessment	15	Describe any methods used to assess certainty (or confidence) in the body of evidence for an outcome.	3-5
**RESULTS**			
Study selection	16a	Describe the results of the search and selection process, from the number of records identified in the search to the number of studies included in the review, ideally using a flow diagram.	6, Figure 1

Section and Topic	Item #	Checklist item	Reported on page #
	16b	Cite studies that might appear to meet the inclusion criteria, but which were excluded, and explain why they were excluded.	N/A
Study characteristics	17	Cite each included study and present its characteristics.	Table 1
Risk of bias in studies	18	Present assessments of risk of bias for each included study.	11, Table S3
Results of individual studies	19	For all outcomes, present, for each study: (a) summary statistics for each group (where appropriate) and (b) an effect estimate and its precision (e.g. confidence/credible interval), ideally using structured tables or plots.	Figures 2-5, Table 1, Table S3
Results of syntheses	20a	For each synthesis, briefly summarise the characteristics and risk of bias among contributing studies.	8-9. Figures 2-5
	20b	Present results of all statistical syntheses conducted. If meta-analysis was done, present for each the summary estimate and its precision (e.g. confidence/credible interval) and measures of statistical heterogeneity. If comparing groups, describe the direction of the effect.	10-17, Figures 2-5
	20c	Present results of all investigations of possible causes of heterogeneity among study results.	16-17, Table S4
	20d	Present results of all sensitivity analyses conducted to assess the robustness of the synthesized results.	16-17, Table S5
Reporting biases	21	Present assessments of risk of bias due to missing results (arising from reporting biases) for each synthesis assessed.	N/A
Certainty of evidence	22	Present assessments of certainty (or confidence) in the body of evidence for each outcome assessed.	17, Table S6
**DISCUSSION**			
Discussion	23a	Provide a general interpretation of the results in the context of other evidence.	18-20
	23b	Discuss any limitations of the evidence included in the review.	18-20
	23c	Discuss any limitations of the review processes used.	18-20
	23d	Discuss implications of the results for practice, policy, and future research.	18-20
**OTHER INFORMATION**			
Registration and protocol	24a	Provide registration information for the review, including register name and registration number, or state that the review was not registered.	3
	24b	Indicate where the review protocol can be accessed, or state that a protocol was not prepared.	3
	24c	Describe and explain any amendments to information provided at registration or in the protocol.	N/A
Support	25	Describe sources of financial or non-financial support for the review, and the role of the funders or sponsors in the review.	18
Competing interests	26	Declare any competing interests of review authors.	21
Availability of data, code and other materials	27	Report which of the following are publicly available and where they can be found: template data collection forms; data extracted from included studies; data used for all analyses; analytic code; any other materials used in the review.	21

From: Page MJ, McKenzie JE, Bossuyt PM, Boutron I, Hoffmann TC, Mulrow CD, et al. The PRISMA 2020 statement: an updated guideline for reporting systematic reviews. BMJ 2021;372:n71. doi: 10.1136/bmj.n71

Table S2. Search strategy through electronic databases**.**

**MEDLINE**	"Daptomycin"[Text Word] AND "Vancomycin"[Text Word] AND ("aureus"[Text Word] OR "MRSA"[Text Word] OR "S. aureus"[Text Word] OR "Staphylococcus aureus"[TextWord] OR "S. aureus"[Text Word] OR "methicillin-resistance"[Text Word] OR "methicillin-resistant"[Text Word])
**EMBASE**	'daptomycin':ti,ab,kw AND 'vancomycin':ti,ab,kw AND ('aureus':ti,ab,kw OR 'mrsa':ti,ab,kw OR 'staphylococcus aureus':ti,ab,kw OR 's. aureus':ti,ab,kw OR 'methicillin-resistance':ti,ab,kw OR 'methicillin-resistant':ti,ab,kw)
**COCHRANE LIBRARY**	"Daptomycin" and "staphylococcus aureus"

Table S3. (a). Quality assessment of studies through a modified version of the Newcastle-Ottawa Assessment Scale.

**Cohort studies**									
**Study**	SELECTION MAX 4				COMPARABILITY MAX 2	OUTCOME MAX 3			TOTAL STAR RATING UP TO 9
	Representativeness of the exposed cohort	Selection of the non-exposed cohort	Ascertainment of exposure	Outcome not present at start of study	Comparability of cohorts at baseline	Assessment of outcome	Follow-up long enough	Follow-up complete	Assessment of bias risk
Rehm et al.	★	★	★	★	★★	★	★	★	**9 Low risk of bias**
Murray et al.	★	★	★	★	★	★	★	★	**8 Low risk of bias**
Weston et al.	★	★	★	★	★★	★	★	★	**9 Low risk of bias**
Usery et al.	★	★	★	★	★	★	★	★	**8 Moderate[a] risk of bias**
Moise et al.	★	★	★	★	★	★	★	★	**8 Low risk of bias**
Claeys et al.	★	★	★	★	★	★	★	★	**8 Low risk of bias**
Arshad et al.	★	★	★	★	★★	★	★	★	**9 Moderate[b] risk of bias**
**Case-control studies**									
**Study**	SELECTION MAX 4				COMPARABILITY MAX 2	OUTCOME MAX 3			TOTAL STAR RATING UP TO 9
	Adequacy of case definition	Cases representative of the population under investigation	Selection of the controls	Selection of the controls	Comparability of cases and controls at baseline	Assessment of exposure	Same method to ascertain cases and controls	Missing rate	Assessment of bias risk
Moore et al.	★	★	★	★	★	★	★		**9 Low risk of bias**

[a] Downgrade due to study design, originally conceived to compare more groups of antibiotics.
[b] Downgrade due to study design, originally conceived to compare ceftaroline with other antibiotics.

Table S3. (b). Definition for the adapted version of the Newcastle-Ottawa Assessment Scale used to the purposes of the present review.

Cohort studies	Criteria to fulfill	
Category: Selection	Representativeness of the exposed cohort	Cases of MRSA BSI/endocarditis exposed to daptomycin
	Selection of the non exposed cohort	Cases of MRSA BSI/endocarditis exposed to vancomycin drawn from the same population of the exposed to daptomycin
	Assessment of exposure	Secure records
	Demonstration that the outcomes of interest were not present at the study start	Yes
Category: Comparability	Comparability of cohorts on the basis of design or analysis	If propensity score matching: two stars
		Other matching methods involving at least criteria among age, severity of illness (e.g. Pitt Score), comorbidity burden (e.g. Charlson Score Index): one star; alternatively, overlapping baseline features between groups
Category: Outcome	Assessment of outcome	Prospective collection of data or record linkage
	Long enough follow-up	At least 30 day
	Adequacy of follow-up	Complete data for all subjects accounted for or limited loss to follow-up (maximum)
Case-control studies		
Category: Selection	Adequacy of case definition	MRSA BSI according to international definitions
	Representativeness of the cases	Consecutive or obviously representative series of cases
	Selection of the controls	Controls from the same population of cases
	Definition of controls	Subjects with MRSA BSI/endocarditis receiving vancomycin
Category: Comparability	Comparability of cases and controls on the basis of design or analysis	Study controls for age and severity of illness (e.g. Pitt Score): one star
		Study controls for any additional factor (e.g., comorbidity burden): another star
Category: Exposure	Ascertainment of exposure	Secure records
	Same method of ascertainment for cases and controls	Yes
	Missing rate	Same for both groups

BSI: bloodstream infection; MRSA: methicillin-resistant *Staphylococcus aureus.* Note: A study can be awarded a maximum of one star for each item as for the Selection and Outcome categories; a maximum of two stars con be given for Comparability.

Table S4. Results of subgroup analyses (mortality as outcome).

	**Included studies**	**Sample size**	**OR, 95% CI**	**I^2**	**Subgroup difference**
Study quality					
• Moderate risk of bias	2	209	2.45 (1.11-5.40)	0%	
• Low risk of bias	6	1017	0.51 (0.30-0.86)	45%	p = 0.001
Endocarditis proportion					
• ≤ 20%	4	689	0.65 (0.31-1.38)	65%	
• > 20%	4	537	0.81 (0.27-2.39)	75%	p = 0.76
Study mortality					
• ≤ 15%	3	539	0.59, 0.16-2.13	76%	
• > 15%	5	687	0.82, 0.39-1.70	67%	p = 0.66
Combination therapy					
• Yes*	6	1017	0.51 (0.30-0.86)	45%	
• No	2	209	2.45 (1.11-5.40)	0%	p = 0.001

* When excluding the study by Rehm et al. [26], OR was 0.44 (95% CI. 0.29-0.66), I^2 equal to 12%, 929 the sample size, and statistically significant the test for interaction (p = 0.0001).CI: confidence interval; OR: odds ratio.

Table S5. Results of leave-one-out meta-analysis (random-effects model).

Outcome: mortality		
**Study excluded**	**Pooled OR (95% CI)**	**Heterogeneity (I^2), %**
Rehm et al.	0.65 (0.34-1.24)	67
Moore et al.	0.80 (0.41-1.56)	69
Murray et al.	0.82 (0.44-1.54)	68
Weston et al.	0.82 (0.42-1.59)	67
Usery et al.	0.63 (0.34-1.16)	64
Claeys et al.	0.81 (0.42-1.59)	67
Moise et al.	0.72 (0.35-1.49)	71
Arshad et al.	0.61 (0.34-1.09)	59
**Outcome: clinical failure**		
**Study excluded**	**Pooled OR (95% CI)**	**Heterogeneity (I^2), %**
Rehm et al.	0.58 (0.36-0.94)	65
Moore et al.	0.61 (0.37-0.98)	65
Murray et al.	0.65 (0.42-0.99)	52
Weston et al.	0.59 (0.36-0.98)	65
Usery et al.	0.53 (0.34-0.81)	57
Claeys et al.	0.60 (0.36-1.02)	65
Moise et al.	0.61 (0.38-0.98)	64
Arshad et al.	0.48 (0.35-0.65)	19
**Outcome: recurrence**		
**Study excluded**	**Pooled OR (95% CI)**	**Heterogeneity (I^2), %**
Rehm et al.	0.89 (0.38-2.12)	22
Moore et al.	1.11 (0.52-2.35)	21
Murray et al.	1.12 (0.64-2.13)	0
Weston et al.	0.83 (0.43-1.62)	0
Usery et al.	1.29 (0.66-1.25)	2
Moise et al.	1.06 (0.56-1.98)	9
Arshad et al.	1.00 (0.49-2.01)	27
**Outcome: persistent BSI**		
**Study excluded**	**Pooled OR (95% CI)**	**Heterogeneity (I^2), %**
Rehm et al.	0.71 (0.36-1.42)	72
Murray et al.	0.96 (0.51-1.81)	61
Weston et al.	0.83 (0.40-1.76)	75
Usery et al.	0.62 (0.36-1.06)	51
Claeys et al.	0.91 (0.42-1.94)	72
Moise et al.	0.73 (0.37-1.47)	74
**Outcome: LOS**		
**Study excluded**	**Mean difference (95% CI)**	**Heterogeneity (I^2), %**
Murray et al.	1.48 (-0.66, 3.63)	17
Usery et al.	0.79 (-1.00, 2.59)	36
Claeys et al.	0.03 (-1.70, 1.76)	0
Moise et al.	1.32 (-0.67, 3.30)	26
**Outcome: Safety**		
**Study excluded**	**Pooled OR (95% CI)**	**Heterogeneity (I^2), %**
Rehm et al.	0.37 (0.09-1.52)	0
Moore et al.	0.14 (0.02-1.01)	31
Murray et al.	0.03 (0.00-0.26)	0
Claeys et al.	0.23 (0.06-0.84)	32
Moise et al.	0.09 (0.01-1.57)	37
**Outcome: 30-day readmission**		
**Study excluded**	**Pooled OR (95% CI)**	**Heterogeneity (I^2), %**
Murray et al.	1.01 (0.58-2.06)	0
Moise et al.	0.87 (0.44-1.71)	0
Arshad et al.	0.94 (0.56-1.55)	0

BSI: bloodstream infection; CI: confidence interval; LOS: length of stay; OR: odds ratio.

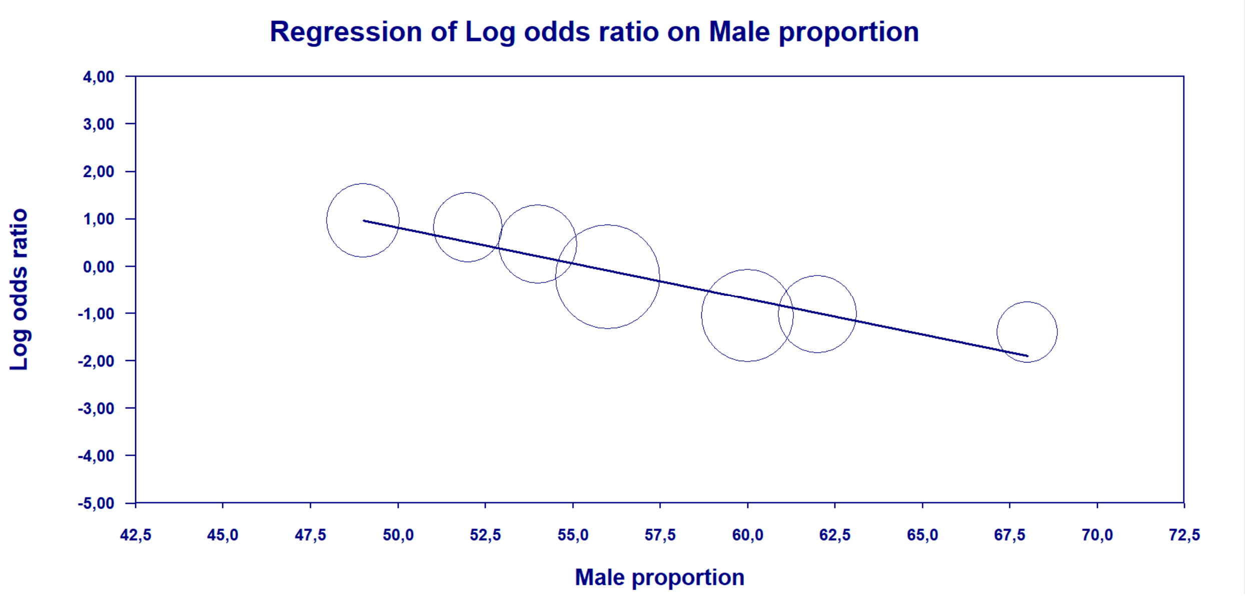

Figure S1. Bubble plot displaying the result of the meta-regression.

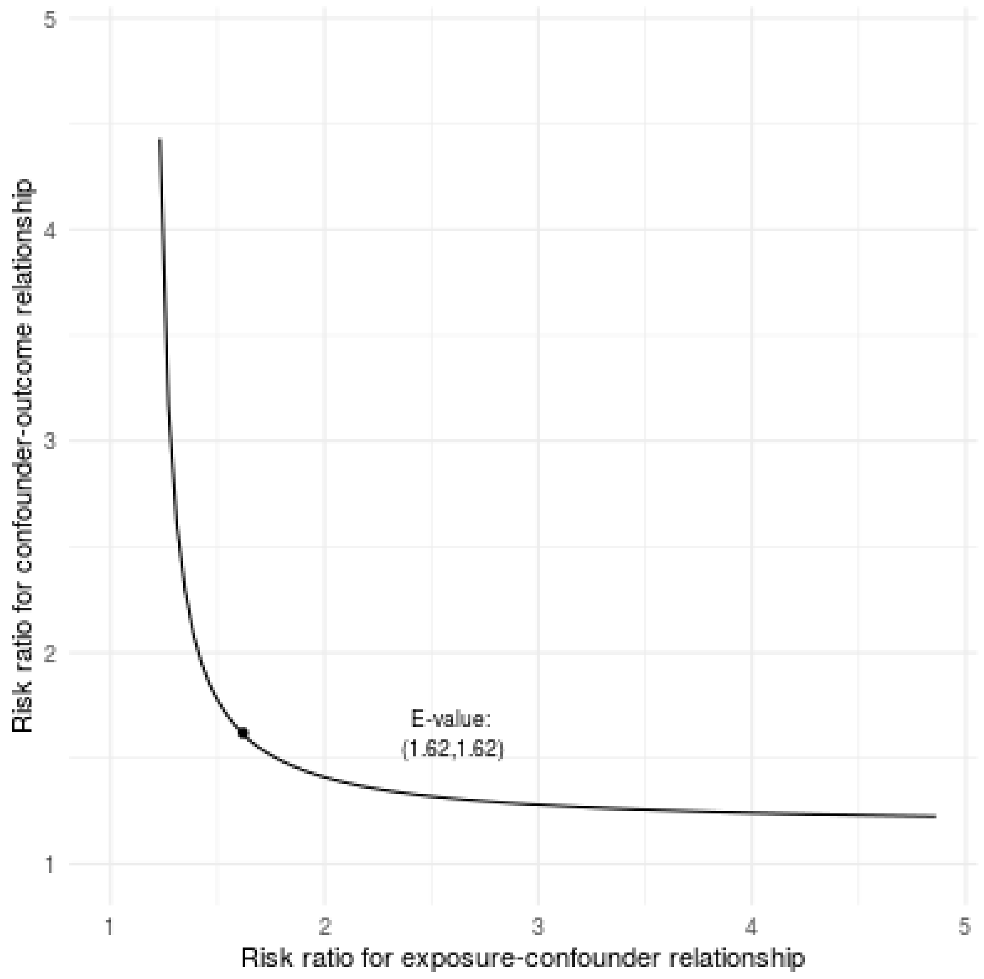

Figure S2. E-value plot.

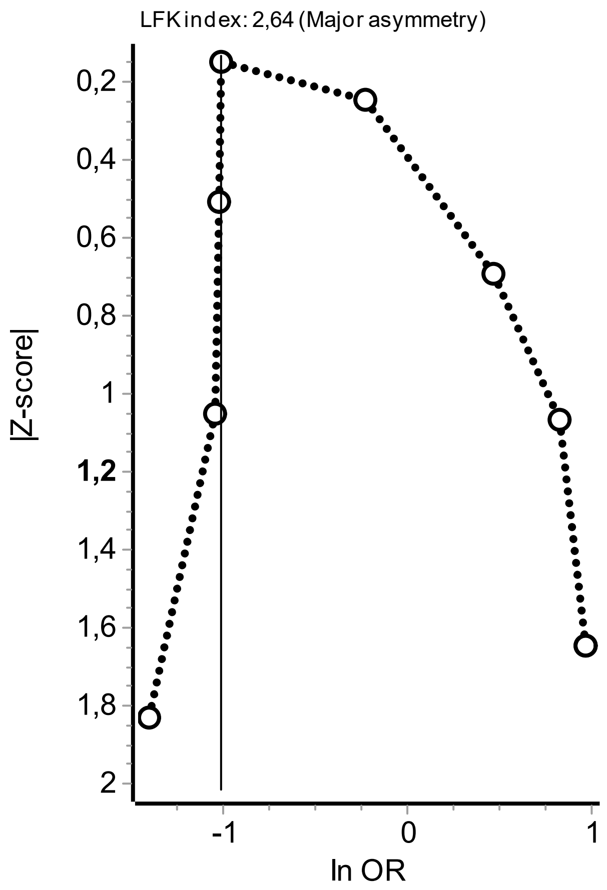

LFK: Luis Furuya-Kanamori.

Figure S3. Doi plot analysis and LFK index of publication bias.

Table S6. Certainty of evidence according to the GRADE framework.

Outcome	No of studies	Risk of Bias	Inconsistency	Indirectness	Imprecision	Publication bias	Certainty of evidence
Mortality	8	Not serious	Serious	Not serious	Not serious	Serious	Low
Clinical failure	10	Not serious	Serious	Not serious	Not serious	Serious	Low
Relapse	7	Not serious	Not serious	Not serious	Serious	Serious	Low
Persistence	6	Not serious	Serious	Not serious	Serious	Serious	Very low
Length of stay	4	Not serious	Not serious	Not serious	Serious	Serious	Low
Safety profile	5	Not serious	Not serious	Not serious	Not serious	Serious	Moderate
Re-admission	3	Not serious	Not serious	Not serious	Serious	Serious	Low

Grade Definitions about certainty of evidence.

High: Further research is very unlikely to change our confidence in the estimate of effect.

Moderate: Further research is likely to have an important impact on our confidence in the estimate of effect and may change the estimate.

Low: Further research is very likely to have an important impact on our confidence in the estimate of effect and is likely to change the estimate.

Very low: Any estimate of effect is very uncertain.
